# Spatial and temporal structure of typhoid outbreaks in Washington, D.C., 1906–1909: evaluating local clustering with the *G*_i_* statistic

**DOI:** 10.1186/1476-072X-5-13

**Published:** 2006-03-27

**Authors:** Sarah E Hinman, Jason K Blackburn, Andrew Curtis

**Affiliations:** 1 World Health Organization Collaborating Center for Remote Sensing and GIS for Public Health, Department of Geography and Anthropology, Louisiana State University, Howe-Russell Geoscience Complex, Baton Rouge, Louisiana, USA

## Abstract

**Background:**

To better understand the distribution of typhoid outbreaks in Washington, D.C., the U.S. Public Health Service (PHS) conducted four investigations of typhoid fever. These studies included maps of cases reported between 1 May – 31 October 1906 – 1909. These data were entered into a GIS database and analyzed using Ripley's K-function followed by the *G*_i_* statistic in yearly intervals to evaluate spatial clustering, the scale of clustering, and the temporal stability of these clusters.

**Results:**

The Ripley's K-function indicated no global spatial autocorrelation. The *G*_i_* statistic indicated clustering of typhoid at multiple scales across the four year time period, refuting the conclusions drawn in all four PHS reports concerning the distribution of cases. While the PHS reports suggested an even distribution of the disease, this study quantified both areas of localized disease clustering, as well as mobile larger regions of clustering. Thus, indicating both highly localized and periodic generalized sources of infection within the city.

**Conclusion:**

The methodology applied in this study was useful for evaluating the spatial distribution and annual-level temporal patterns of typhoid outbreaks in Washington, D.C. from 1906 to 1909. While advanced spatial analyses of historical data sets must be interpreted with caution, this study does suggest that there is utility in these types of analyses and that they provide new insights into the urban patterns of typhoid outbreaks during the early part of the twentieth century.

## Background

From the mid-nineteenth century until the turn of the twentieth century, germ theory gradually overtook miasmatic theory as the dominant understanding of disease causation [1,2]. During this period, many large cities in the United States continued to suffer from epidemic cycles, nation-wide epidemic diffusions, and almost annual *in situ *outbreaks of disease. For example, regular typhoid fever outbreaks continued to occur in Washington, D.C. throughout the early twentieth century. In an attempt to understand these epidemic occurrences, many city and state board of health reports contained detailed spatial epidemiological descriptions of disease genesis and progress. These reports were often accompanied by cartographic displays, and in some instances cartographic overlays, providing a means of comparing multiple spatial layers to gain insights into disease distribution [3]. This early form of spatial analysis allowed health officials and policy-makers to frame mitigation strategies for subsequent years. The quality of many of these data sets is suitable for further spatial analytical investigations utilizing new geospatial statistics and software applications.

The quantitative analysis of historical disease patterns is not a new field of inquiry. Examples range from the collapse of Amerindian society through smallpox [4,5], cholera in the Philippines at the turn of the twentieth century [6,7] as well in the United States during the late nineteenth century [8], yellow fever in New Orleans (1853) [9,10], the spatial structure of the 1916 poliomyelitis epidemic [11], to typhoid in 1898 in the United States [12]. These last two papers provide comparable approaches to this current research in terms of data sources and an analytical approach. A contemporary report of a typhoid epidemic was used to further a general understanding of disease diffusion [13,14]. In the poliomyelitis example the authors applied spatial autocorrelation techniques, including the G_i_(d) variant of the Getis and Ord statistic, to understand geographic patterns in their historical dataset [11]. The contemporary datasets used in both studies came from reports produced by the United States Public Health and Marine-Hospital Service (PHS). More specifically the typhoid report used by Smallman-Raynor and Cliff [12] forms part of a series of PHS studies that were conducted to better understand the distribution of typhoid in susceptible populations. The study presented here utilized typhoid reports for Washington, D.C. from the same series as those used by Smallman-Raynor and Cliff [12].

The application of geographic information system (GIS) technologies to city-level datasets contained in many late nineteenth and early twentieth century reports is warranted due to their spatial richness, which was often at the residence level. The justification for such research is to gain a fresh understanding of the spatial epidemiology of a specific disease [[11], p. 701–702], as well as adding insight into urban epidemic structure, and a fresh historical perspective on how the urban environment can affect disease patterns. When multiple years are analyzed, the relationship between urban infrastructure change and the impact of post-epidemic mitigation strategies on disease surfaces can also be analyzed. This paper will specifically focus on the spatial epidemiology and urban epidemic structure of typhoid fever for the period 1906 – 1909. Future research using this dataset will explore the urban environmental aspects and infrastructure change elements associated with an urban epidemic.

Spatial analytical techniques and models are often used in epidemiology to identify spatial anomalies (hotspots) in disease surfaces. These analytical approaches can be used to not only identify the location of hotspots, but also their spatial structure. This paper uses two such spatial analytical approaches by applying a Ripley's K-function, a measure of global spatial autocorrelation, followed by the *G*_i_* statistic, a method of local spatial autocorrelation, to four consecutive typhoid outbreaks in Washington, D.C. (1906 – 1909). These statistics, which have been used in recent epidemiological investigations to determine spatial cluster size for arthropod-vectored diseases were used here to identify the location and size of disease hotspots across Washington, D.C. during the four outbreaks [15-17]. These approaches can be used to not only reveal the spatial structure of a single epidemic, but can also be combined across the years to identify temporal stability in disease presence.

### Typhoid fever

Typhoid fever is caused by *Salmonella typhi*, a bacterial species endemic to humans. The bacteria is carried in the bloodstream and gastro-intestinal tract of infected persons and shed through feces. The disease is media-borne and most often transmitted through contaminated water or food, especially in parts of the world where water treatment and sewage infrastructure are limited [16]. Typhoid infection, which is also called typhoid fever because of the high fever often associated with the disease, includes other symptoms such as stomach pain, headache, weakness, loss of appetite, and nausea [18,19]. Typhoid remains a minor problem in the United States with approximately 400 cases appearing annually, although about 70 percent of these are contracted in other countries [18].

Typhoid, as with other diarrheal diseases such as cholera and dysentery, often produces interesting geographic patterns due to its diffusion through a shared water supply. Many introductory geography classes use the example of John Snow as an exploratory spatial investigation. The typical classroom description of Snow's work describes how the plotting of cholera cases led to the identification of a disease focus and an intervention strategy – the removal of the Broad Street water pump handle in 1854 London [20]. The degree to which this spatial visualization of the disease resulted in a solution to the outbreak is now in doubt, as it is believed the map was created some time after the outbreak and merely served to explain the spatial logic behind Snow's approach. The fact remains, however, that a spatial determination of the disease surface was attempted [20,21].

Although most frequently cited, John Snow's work is not the only example of spatial analyses being applied to now historical disease surfaces. For example, William Sedgwick's investigation of a typhoid epidemic in Lowell and Lawrence, Massachusetts in 1890 and 1891 used a similar "shoe-leather epidemiology" and cartography approach. Sedgwick studied all of the reported cases of typhoid along with the different water supplies to the cities from which the cases might have developed. He ultimately traced the origin of the epidemic to a source of infection in the upriver town of North Chelmsford. From there, the bacteria traveled down the Merrimac River to drinking water intakes in Lowell and Lawrence. As a result of this study of typhoid, Lowell ceased to draw drinking water from the Merrimac River and Lawrence built a water filtration plant. These mitigation techniques significantly reduced typhoid fever outbreaks in both cities [22]. Current computational and spatial analytical advances allow such disease surfaces to now be further investigated in order to gain a better understanding of disease patterns and processes [11].

### Typhoid in Washington, D.C

The city of Washington, D.C. opened a new water supply system in 1905 intending to reduce water-borne disease outbreaks. However, during June 1906 a typhoid epidemic erupted in the city resulting in a higher morbidity rate than before the 1905 infrastructural improvements. As an outcome of this situation the city commissioned the PHS to investigate the origins of the epidemic, and simultaneously conduct a bacteriological study of the water from all public pumps in the city. The PHS chose to continue annual investigations of typhoid until 1909, when the typhoid rate in Washington dropped to acceptable levels for a city given its sanitary infrastructure [23]. The PHS reports were submitted to both the U.S. Congress and the city of Washington, as at this time, Congress oversaw the administration of Washington, D.C. Interestingly, in the original request for help to the PHS, direct reference was made to the apparent uniform spatial distribution of the cases during the typhoid outbreak [[24], p. 10]. This suggested a mass consumption source of the disease rather than localized source of infection. As no immediate spatial pattern was discernable, the Health Officer of the city of Washington primarily charged the PHS to find the epidemic's source. In following this directive, PHS doctors investigated the geographic distribution of the disease; the result was a series of maps in each report displaying the location of each typhoid case studied [23-26]. These maps, supported by the accompanying descriptions in the reports, have now been transferred into a GIS allowing for more sophisticated spatial analysis – in effect finally meeting the mission charged of the PHS.

The spatial analytical capabilities of GIS now allow for these four typhoid epidemics to be further investigated in the form of three general research questions. First, was the spatial distribution of typhoid in Washington, D.C. truly uniform, as suggested in the original reports, or did clusters of disease exist within the city? Second, if clusters were present, at what spatial scale did they exist – highly localized, or generalized across large parts of the city? General city-wide epidemics would generate large spatial clusters with relatively homogenous morbidity surfaces. Although concentrated disease clusters would likely occur as secondary sources of infection, the ratio of these to large clusters would diminish during city-wide epidemics. Third, did these clusters remain temporally stable, with individual hotspots existing across multiple outbreak years? As the socioeconomic characteristics of neighborhoods were unlikely to change dramatically between years, any general source of infection, especially without specific intervention, would likely impact similar areas of the city annually. Additionally, if a small disease cluster were to appear for numerous years, this might reveal an underlying and consistent disease threat, such as an unsanitary well or a small manufacturer with poor hygiene facilities.

### Data quality

As previously stated, many health reports originating from the late nineteenth and early twentieth centuries contain spatial data, usually in the form of tables, lists, and maps, suitable for investigation using a GIS. However, before embarking on a GIS analyses it is necessary to understand the limitations of these types of data [27]. It has previously been suggested that three requirements are needed in order to be confident about historical disease data; the disease had to have been well understood at the time of data recording, cases could be clinically defined, and there was an officially recognized reporting mechanism [28]. All of these criteria are met by the data under investigation in this study.

It is also usually preferable to analyze mortality rather than morbidity data due to confounding similarities in the early onset symptoms of many diseases [29]. However, as medical care improved in the nineteenth and early twentieth centuries a shift occurred in the ratio between morbidity and mortality events. The simple understanding of the importance of bedside care and cleanliness meant that mortalities became increasingly skewed to the more vulnerable populations, such as the very young and the elderly. In relying on mortality data, resulting spatial cluster patterns might reflect the distribution of the susceptible cohort rather than the underlying disease surface. Therefore, morbidity data, even given the limitations of symptom similarity, does provide a more holistic spatial impression of the outbreaks [30,31].

Typhoid offers an additional problem in terms of accurately linking all cases to a single origin in a temporal sequence, as an eventual death could take months after the initial infection. For this reason, some health reports in the late nineteenth century administered surveys where attempts were made to identify the initial onset of symptoms as this could be used as a spatial linking mechanism [23-26,32]. Other data problems could occur, especially during severe epidemics in which physicians would often take flight leaving laypeople to diagnose and complete official mortality documents [33]. In addition, standard reporting problems to any health investigation, both current and past, would have to be faced. These include omissions or errors in reporting, like nativity or age, and of particular importance to space-time analyses, the accurate and precise recording of morbidity and mortality events by location and date [34]. The presence of these data problems, which are largely impossible to counter, must temper any interpretation of results. Though as long a no systematic bias is present, results should still provide relevancy if the data are considered to be at worst an adequate sample taken from the disease situation.

## Results

**Figure 1:**
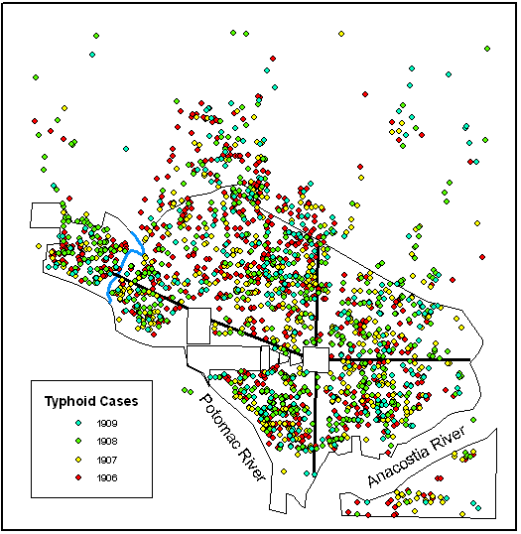
All cases of typhoid that showed signs of onset between 1 May – 31 October for all years studied (1906–1909).

**Figure 2:**
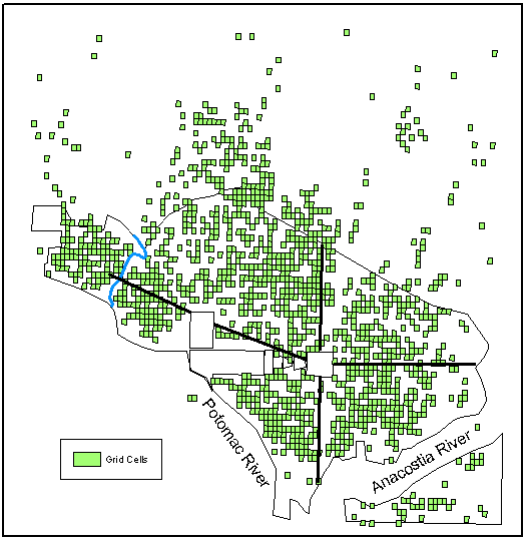
The distribution of the 90 m × 115 m grid cells with one or more typhoid cases from any one of the years studied used in the *G*_i_* analysis.

Figure 1 illustrates the original distribution of point data for each of the four years that was used for each of the K-function analyses. Figure 2 illustrates the grid cells used in the *G*_i_* analysis and represent the grid cells referenced in the individual results sections.

### Ripley's K-function

The global spatial autocorrelation statistic, , indicated a lack of global typhoid clustering in all four years. Table 1 summarizes the results of this analysis. The values for  were fairly similar at all distances in all years studied.

**Table 1:** Ripley's K-Function results.

Distance (m)	1906	1907	1908	1909
0	192.88	197.80	216.99	170.77
100	350.24	368.19	381.27	312.82
200	495.41	511.65	534.79	457.20
300	639.88	644.29	681.38	603.48
400	772.84	789.88	833.06	729.71
500	905.25	914.98	974.53	858.60
600	1036.84	1047.79	1098.68	973.26
700	1160.23	1177.82	1226.26	1090.83
800	1279.21	1304.02	1350.98	1207.75
900	1404.37	1423.07	1468.40	1330.13
1000	1525.93	1535.74	1580.81	1438.93

### *G*_i_* – 1906

**Figure 3:**
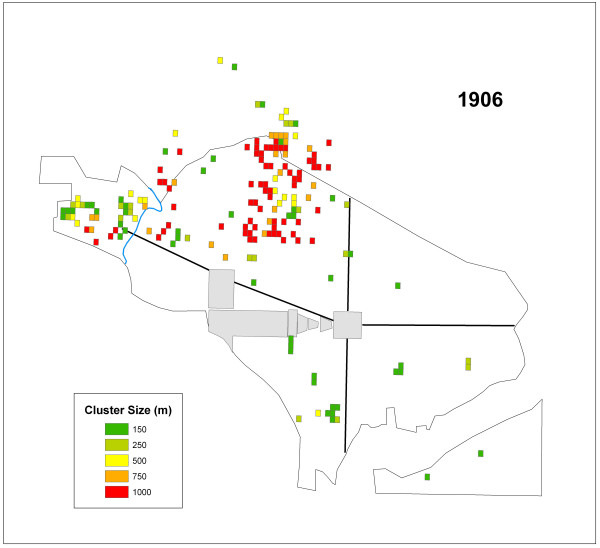
All of the cells representing significant clusters in 1906 displayed by their cluster size.

**Figure 4:**
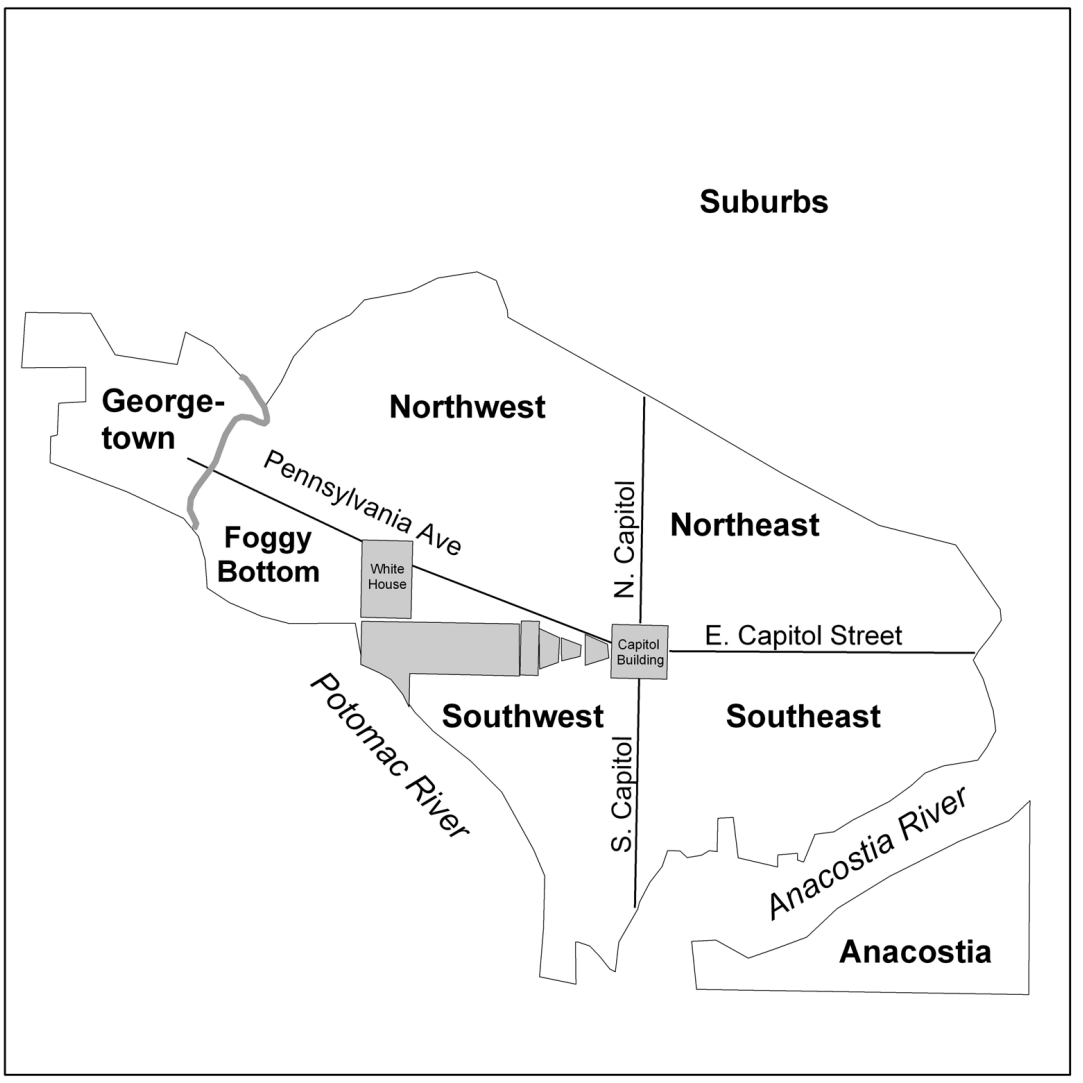
To simplify discussion on the different parts of the city, the city was divided into eight areas based on the common way in which these city regions were, and are, referred to by the local population.

The number of cells that were significant at each distance value for 1906 are summarized in Table 2. The skewing towards the largest cluster size suggested a broad geographic distribution of infection. Figure 3 displays significant grid cells by cluster size for this year. All but one of the clusters in Southwest were at more localized scales (150 m, 250 m), while most of the 1000 m clusters appear in the Northwest near the northern boundary (Figure 4).

**Table 2:** Summary of the *G*_i_* analysis by individual years.

Year	Distance (m)	Cells	mean *G*_i_*	min *G*_i_*	max *G*_i_*
*1906*					
	150	47	2.80	2.03	4.99
	250	20	2.62	2.03	3.53
	500	20	2.61	2.01	3.53
	750	22	2.58	2.12	3.27
	1000	69	2.57	2.00	4.06
*1907*					
	150	40	2.79	2.03	7.52
	250	28	2.90	2.11	4.47
	500	36	2.69	2.02	3.67
	750	26	2.71	2.03	3.56
	1000	21	2.44	2.04	3.29
*1908*					
	150	23	3.00	2.22	5.10
	250	19	2.70	2.01	4.61
	500	17	4.42	2.03	7.09
	750	30	4.32	2.00	5.76
	1000	69	3.62	2.02	5.18
*1909*					
	150	32	2.73	2.21	4.86
	250	19	2.95	2.00	4.70
	500	14	2.86	2.04	4.17
	750	11	2.43	2.13	2.95
	1000	9	2.51	2.10	2.90

### *G*_i_* – 1907

**Figure 5:**
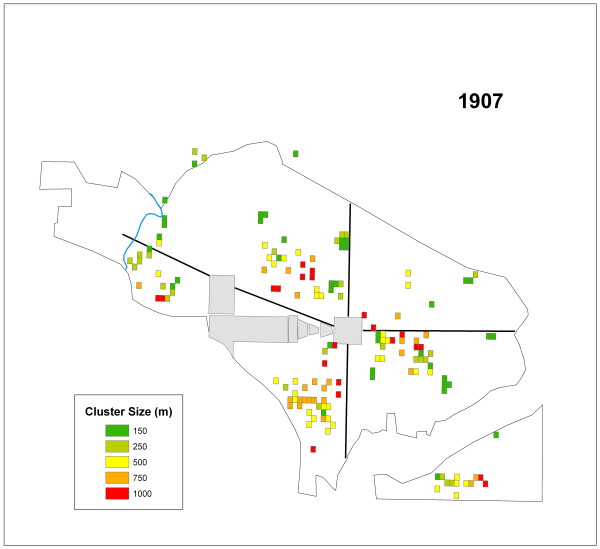
All of the cells representing significant clusters in 1907 displayed by their cluster size.

The summary of the number of cells that were significant at each distance value are reported in Table 2. Figure 5 displays the significant grid cells by cluster size for 1907. Interestingly, one single cell had the highest overall *G*_i_* value of 7.5. This cell, with a maximum cluster distance of 150 m suggests a strong localized outbreak.

### *G*_i_* – 1908

**Figure 6:**
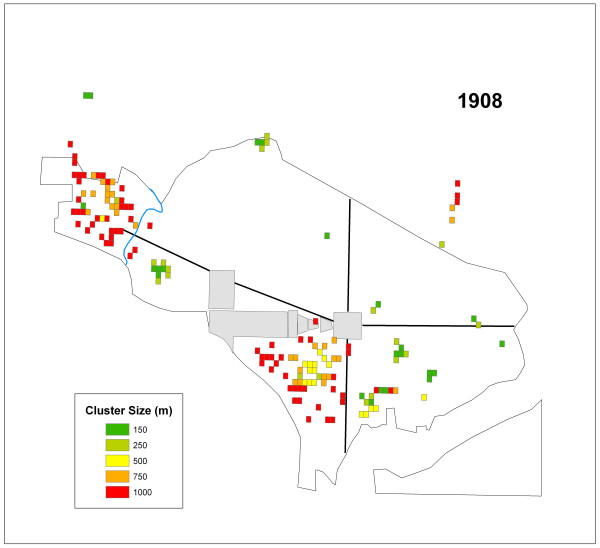
All of the cells representing significant clusters in 1908 displayed by their cluster size.

Table 2 summarizes the number of cells that were significant at each distance value in 1908. Figure 6 displays the significant grid cells by cluster size for this year. It is evident that two sections of the city were primarily impacted during 1908, and these areas tended to display clusters at larger distance values. Three tight groups of localized clusters are visible, as well, in Foggy Bottom, Northwest, and Southeast (Figure 4). Like 1906, significant cells were skewed toward the 1000 m distance value.

### *G*_i_* – 1909

**Figure 7:**
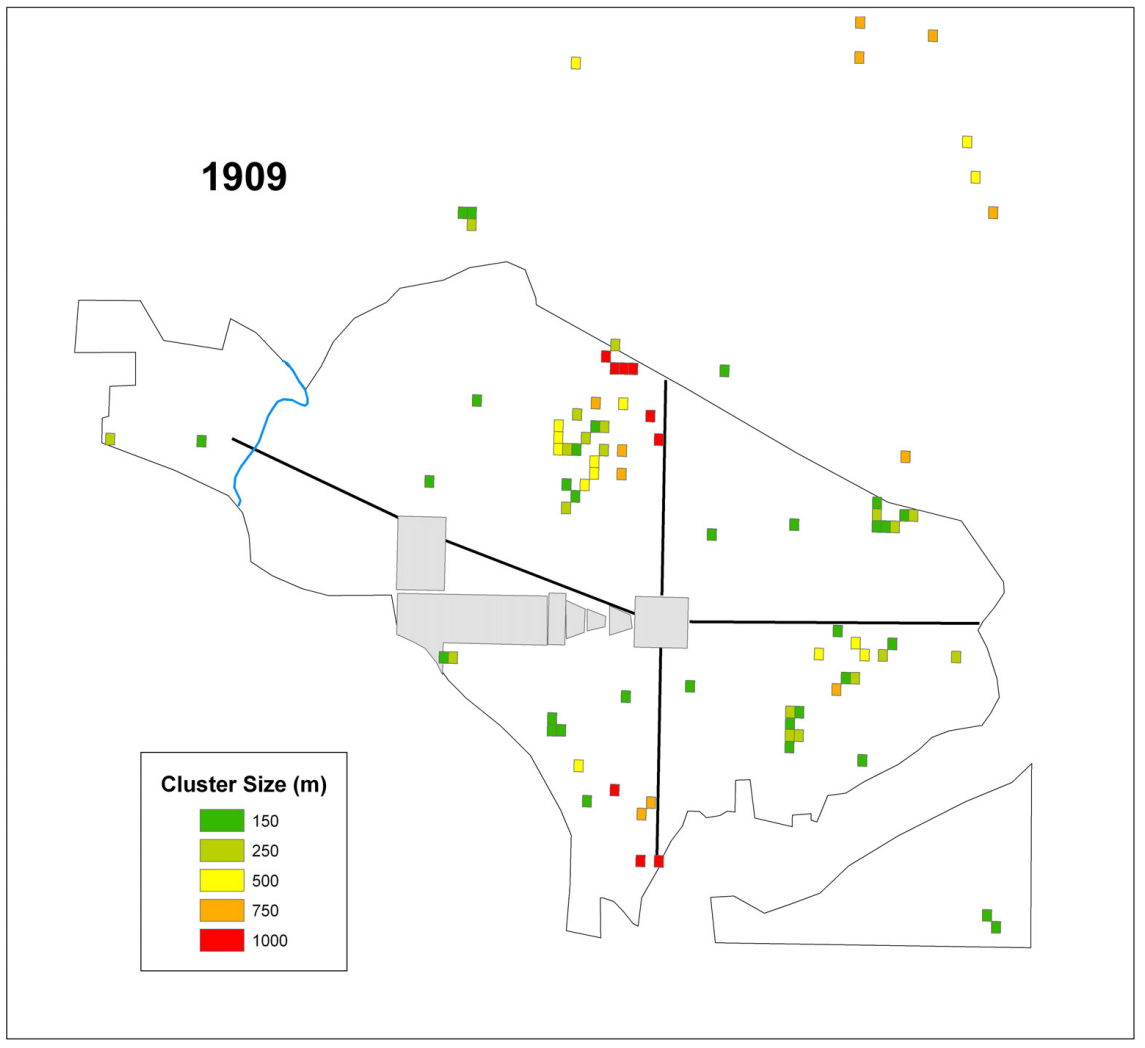
All of the cells representing significant clusters in 1909 displayed by their cluster size.

The number of cells that were significant at each distance value for 1909 are summarized in Table 2. Figure 7 displays the significant grid cells by cluster size for 1909. This year displays more significant cells at the 150 m distance than any other distance explored here. The significant cells, regardless of cluster size, are more dispersed in 1909 than the other years.

### Temporal results

To evaluate temporal stability in spatial hotspots between years, in other words to identify city areas that were conducive to typhoid in all time periods, the total number of years any cell had a significant *G*_i_* value at each distance were tallied. All of these tallies are displayed in Table 3. Interestingly, at all distances most cells were only significant in a single year. Only five cells were significant in two years, and no cells were significant at a particular distance for more than two years. When significant clusters are considered regardless of distance, most cells were still only significant in one year. Only two cells were significant in three years, and no cells were significant cluster members for the duration of the study period regardless of cluster size.

**Table 3:** The total number of years any given grid cell was a member of a spatial cluster at specific distances(d) and for any given distance based on the *G*_i_* values.

	Number of cells significant in years				
Distance (m)	1	2	3	4	Total
150	138	2	0	0	140
250	86	0	0	0	86
500	87	0	0	0	87
750	87	2	0	0	89
1000	165	3	0	0	168

Number of cells significant regardless of distance	482	42	2	0	526

## Discussion

The K-function analysis indicated a lack of global clustering. While this result for each year in the study period supports the conclusions of the original reports it does not indicate that clustering was non-existent in the city. The hypothesis, that typhoid clustered locally, was supported in the results of the *G*_i_* statistic. The *G*_i_* statistic results also demonstrated that the disease clustered at multiple scales. These findings refute the conclusions drawn in all four PHS typhoid reports concerning the distribution of cases where the only mention of possible large concentrations of typhoid cases was within the 1908 report. Results of this current study indicated both widespread and localized clusters occurred in all four years (Table 2; Figures 3, 5, 6, and 7).

The second question addressed whether or not clusters varied in size during each year. Again, variation existed in the ratios of cluster sizes within and between the outbreak years. Two years, 1906 and 1908 skewed toward clusters of 1000 m suggesting that large areas of the city were infected over the course of the study period. However, 1907 and 1909, showed a more localized pattern of infection, with more clusters at a critical distance of 150 m in these years.

When assessing the question of temporal stability across the study period, we can consider each region of the city separately (Figure 4). Each region of the city appears to have had varied degrees of typhoid intensity within and between outbreak years generally indicating a potential lack of a consistent source of the bacteria (Tables 2 and 3).

Georgetown was impacted in 1906 and 1908, with moderate to large clusters, lacked cases in 1907, and saw only four localized clusters in 1909. The 1908 milk related outbreak in this area was investigated by Rosenau and his colleagues in order to find the source of a rapid increase in typhoid cases at a time when cases had been decreasing that year [26]. Through the investigation, the PHS officers found that only households purchasing milk from two different dairymen during September 1908 were reporting new typhoid cases. Other households on the same streets using different milk suppliers did not report new typhoid cases. The detailed investigation by the PHS narrowed the possible sources of typhoid carried into the city through milk to a single dairy in Maryland, which supplied both dairymen on a daily basis. The owner of the Maryland dairy was a typhoid carrier, having contracted the disease 18 years previously, and although not displaying symptoms since that time was still shedding bacteria. Following the discovery of a typhoid carrier at a dairy, the sale of milk from that dairy was discontinued and eight days later the last case along the routes of the two milk suppliers was reported [26].

Foggy Bottom was most dramatically impacted in 1907. In 1908 this area displayed a tight group of localized clusters, and no clusters in 1909. Between 1900 and 1920 a small African-American residential enclave had developed in this part of the city. Since more black residents contracted typhoid than white residents it is plausible that there would be a disproportionately high number of clusters in these neighborhoods [26,35]. This geographic variation associated with race is at least partly explained by poverty and a majority of the city's alleys, unsanitary or otherwise, being home to African-Americans rather than whites [35-38].

Northwest was most notably impacted in 1906, though numerous significant but generally smaller clusters were also evident in 1907 and 1908. This region displayed six localized clusters in 1908, five of which were grouped together at the northern boundary of the city, another predominately African-American area [35]. Northwest was home to the city's elite. As an example of how affluence impacted disease presence, only, one grid cell, and then only in one time period (1906) was significant within a three-block area around Dupont Circle. Other social groups lived in Northwest as well, but these other groups were mixed into the residential structure by residing in alleys behind many of the wealthier households [36,38].

Northeast displays few significant clusters in any of the years. This is likely to be related to the more limited residential settlement in this area [39]. In 1906, 1908, and 1909 these clusters were small, 150 m, and 250 m. Two 1000 m clusters appeared in 1907 close to the Capitol Building. The smaller and more diffuse population in Northeast could help to explain the more localized clusters in this region.

Significant clusters existed in Southeast in all four years, with the greatest number of significant cells occurring in 1907 and 1908. These years also included more clusters at larger distance values, as well. The loose group of clusters near East Capitol Street in 1907 fell within a neighborhood called Capitol Hill. This residential neighborhood was home to a stable middle class community beginning in the 1870s [39].

Southwest was impacted in all four years. This region of the city was notorious for its poverty, and high rates of disease throughout much of the city's history [38]. The PHS report claimed that in 1908 there were more cases of unknown source in the Southwest than in the north of the city [26]. Figure 6 displays large typhoid clusters in Southwest, while in comparison Northwest displayed only six 150 m and 250 m clusters that year. The report attributed the clusters of typhoid in Southwest to poor sanitary conditions. The relationship between the widespread distribution of typhoid in Southwest compared to the localized clusters Northwest in 1908 helps to support the conclusions of Rosenau et al. that a lack of sanitation may have been at work in that part of the city [26]. If the conditions in Southwest were linked to poor sanitation it is likely the disease situation would have reoccurred across the years. Indeed, an outbreak from any other location in the city could possibly lead to secondary outbreaks within this area through its underlying infrastructure, as long as route ways such as worker movement, existed between the two locations. The precise determination of what constituted poor sanitation from the perspective of the PHS doctors is unclear, but there is good evidence from other secondary sources that Southwest housed residents of lower socio-economic status regardless of race [37]. Additionally, the largest enclave of African-American residents in the city lived in the area bound by the Capitol grounds to the north and extending about four blocks to the west from South Capitol Street (Figure 4) [35-37]. Nonetheless, a more detailed analysis of the social and environmental geography of the city at the time would be needed to accurately address this matter.

The general lack of consistent reoccurring clusters between outbreak years indicates a lack of disease location stability as indicated by Figures 3, 5, 6, and 7 and Table 3. These shifting areas of disease intensity are suggestive of a lack of a common and universal source of infection. Between the visual instability of typhoid clusters in Figures 3, 5, 6, and 7 and a general understanding that by 1906 most of Washington, D.C. had the expected levels of sanitary infrastructure to prevent typhoid fever, it is logical that the disease would not remain spatially stable between years. Instead, it seems that the patterns produced here indicate that typhoid affected different communities and possibly households, indicating a need to better understand both neighborhood dynamics, and more importantly the specific practices of each individual household in order to understand the distribution of typhoid. This latter piece of potentially useful information would be extremely hard to generate given that the data are over a century old. Nonetheless, one could state that neighborhood level interconnections were more likely to be causative than any overall city-level sanitary conditions. This conclusion was also alluded to in the reports themselves though, somewhat obscurely, while describing the disease surface as uniform.

All four typhoid reports (1906 – 1909) attributed between 30 and 50 percent of the cases to contaminated milk, contact with a person carrying the disease, or to contracting the disease outside of Washington, D.C. The source of infection for the remaining cases was unknown. Given the "uniform distribution" of the disease as described by the PHS, the water supply was suspected as the source of infection by the investigators [[24], p. 10]. This hypothesized source of typhoid was particularly favored since over 90 percent of those individuals who contracted typhoid in any of the years studied regularly drank un-boiled tap water. With this idea in mind, and as part of their mandate, the PHS tested water related to the city's supply at all stages from the Potomac River, through the various settling basins and filters, to household taps. The results of these bacteriological investigations were inconclusive in 1906 and during the remaining years continued to be considered "free" of contaminants [23].

Although the overall finding of three reports was that the city's water supply was not the source of infection for cases of unknown cause, a few more specific statements about the distribution of typhoid were made in 1906, 1908, and 1909. The 1906 report included a geographic study of shallow and deep well locations compared to the location of typhoid cases of unknown cause. The conclusion of this particular study was that there was no unusual concentration of cases around well locations. The condition and water quality in wells were not included in the subsequent reports [24,25].

While all four reports described a fairly general distribution of typhoid, the 1908 report made direct reference to two specific concentrations of typhoid cases, the milk related outbreak in Georgetown and the group of cases of unknown cause in Southwest, both discussed above [26]. In 1909, the report summarized the findings from all four years and discussed the possible associations between the disease and a contaminated water supply [23]. The PHS remained reluctant to state that the water supply was the source of the continued presence of typhoid since not only was the disease nearly uniformly distributed, but this distribution included households not hooked up to the city water system. Additionally, in 1909 the PHS investigators mapped the distribution of diphtheria and scarlet fever, two diseases not associated with water, and found a general and uniform distribution of these diseases that resembled the distribution of typhoid. Given these three disease distributions, the authors believed that some mechanism besides water was at work in distributing typhoid around the city [23]. It should be remembered, however, that these distributions were purely visual and not the result of any spatial analytical approach.

The spatial analysis of the datasets contained in the PHS reports for the years 1906 to 1909 should only be seen as the first step in investigating the disease surfaces of early twentieth century Washington, D.C. Further temporal precision can be built into the cluster analysis. In addition, other spatial layers, such as urban and social structure, can be overlaid in a search for a neighborhood association with cluster location.

## Conclusion

The methodology applied in this study was useful for evaluating the spatial distribution and inter-annual patterns of typhoid outbreaks in Washington, D.C. from 1906 until 1909. Ripley's K-function supported the original PHS reports, but recent advances in local spatial autocorrelation techniques allowed this study to go beyond simply a global statistic and explore the possibility of local clusters not apparent in the global patterns. The Getis and Ord statistic indicated that clustering occurred at multiple spatial scales and refuted the original PHS conclusions that typhoid's distribution was evenly distributed. While analyses of historical data sets must be interpreted with caution, this study does suggest that there is utility in these types of analyses, and provides new insights into the urban patterns of a series of typhoid outbreaks. Further work is needed to identify the stability of clusters within years, but this study and its confirmation of localized clusters is a first step towards that end. Additionally, future research using this dataset will explore the urban environmental aspects and infrastructure change to gain further understanding of the spatial interconnections of the neighborhoods in the epidemic cycle.

## Methodology

**Figure 8:**
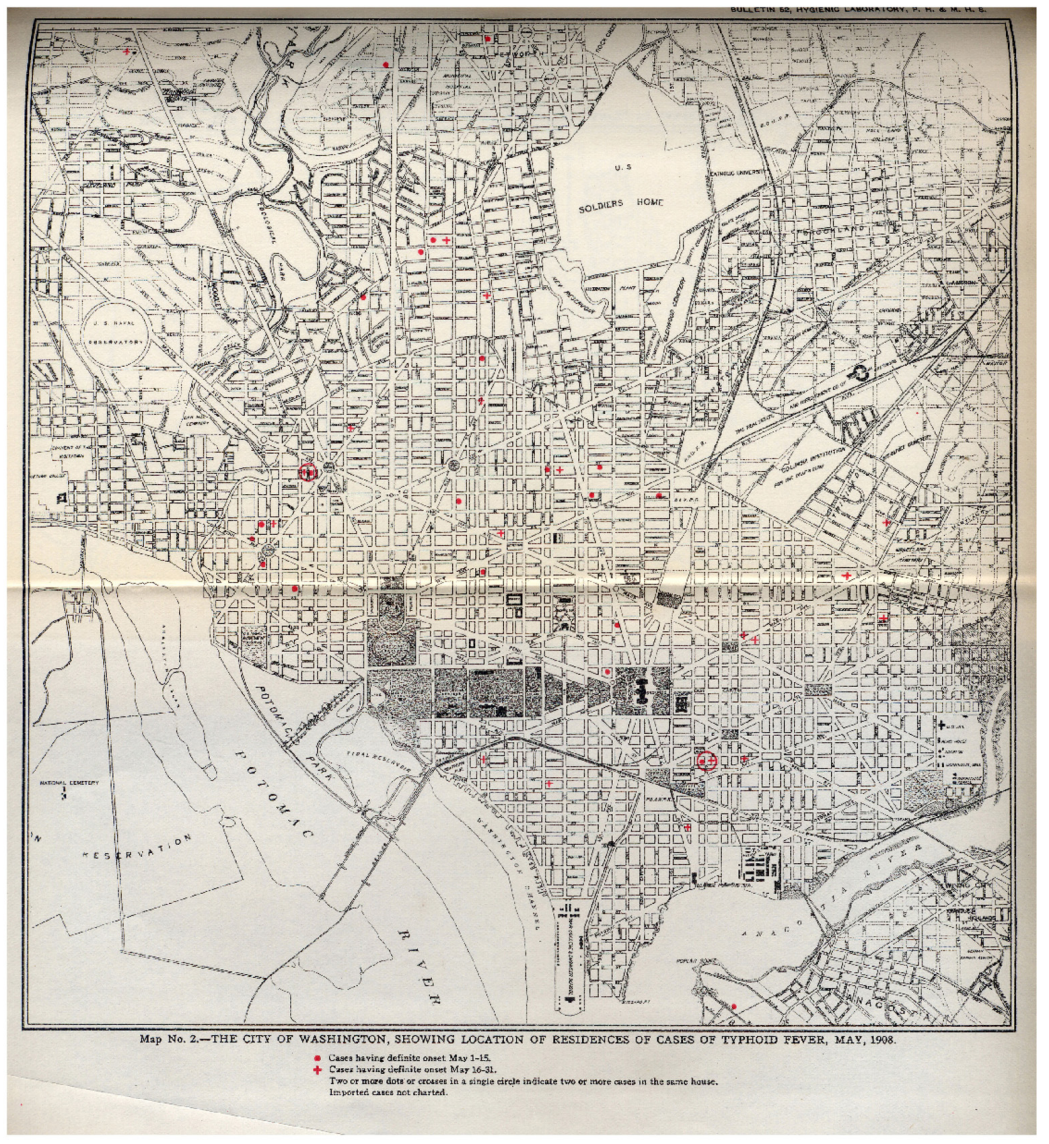
An example of the original map containing typhoid cases. This scanned image is from the 1908 report and displays all cases that showed signs of onset in May.

The typhoid dataset was derived from georeferenced locations of individual disease events extracted from 24 maps contained in the four PHS reports. Each typhoid case was heads-up digitized over an 1898 map of Washington, D.C. georeferenced in ArcGIS 9.0 (Figure 8). The original maps from 1906, 1907, and 1908 showed cases displaying definite symptom onset for each two-week period between May and October of those years. In other words, all cases that developed between 1 May and 15 May were mapped using a different symbol than those cases that developed between 16 May and 31 May. The reports contained separate maps for each of the 6 months. In the process of digitizing these data, each two-week period became its own spatial layer. In 1909, the typhoid cases were mapped for the entire year at the monthly level in the original report, therefore each month between May and October was mapped as its own layer. In order to maintain temporal consistency, the two files generated for each month in 1906, 1907, and 1908 were merged into a single layer. Finally, all six layers from each year were merged together so that this project could evaluate annual cluster stability. The result was four layers of typhoid cases, one for each year used in the study.

### Ripley's K-function

The Ripley's K-function was used to measure global spatial autocorrelation with the original point data for each year. The Ripley's K-function was conducted using ClusterSeer2 (Terraseer, Inc, Crystal Lake, IL). The K-function is written as (following Durbeck et al. [40]):



Where R is the area of the region interest (in this case the defined study boundary- Metropolitan Washington, D.C. and the surrounding "suburban" areas; Figure 4), n is the total number of typhoid cases within the study area (R), d_ij _is the distance between cases *i *and *j*, and I_h_(d_ij_) is an indicator function that equals 1 if d_ij _is less than *h*, and equal to 0 otherwise. Clusterseer employs w_ij _as an edge correction factor (defined from 0.5 to 1) that ensures that cases near the study boundary are evaluated equally [40]. Clusterseer employs a second formula to evaluate the K-function in comparison to a homogeneous Poisson distribution, described as L(h). That formula is expressed as [40]:



We used 10 distance steps from 100 to 1000 m and 1000 Monte Carlo simulations to evaluate L(h) in comparison to Complete Spatial Randomness.

### *G*_i_* statistic

In order to test for statistically significant local typhoid clusters for each year, and to determine the spatial extent of these clusters, the Getis-Ord *G*_i_* statistic was used [41,42]. The *G*_i_* statistic is useful for identifying individual members of local clusters by determining the spatial dependence and relative magnitude between an observation and neighboring observations [15]. The *G*_i_* statistic is written as (following Getis and Ord 1992 [41]; Wu et al. 2004 [43]):



Where x is equal to the number of typhoid cases within a given grid cell, *S *is the standard variance of typhoid cases, when the distance from grid cell *j *to grid cell *i *is within distance *d*. A weights matrix is derived where *w*_*ij*_*(d) *= 1; otherwise *w*_*ij*_*(d) *= 0 to determine whether cases at *j *are within distance *d *of case *i *[43]. The *G*_i_* statistic includes the value at *i *in the calculation of *G*_i_*. *G*_i_* is calculated and then output as the standard normal variant with an associated probability from the z-score distribution [34].

The *G*_i_* is a group-level statistic, where point data must first be aggregated to areas. An 115 m × 90 m vector grid surface was developed to aggregate the spatial distribution of the point data set using the National Park Service Grid Tool extension for ArcGIS 9.0 [44]. *G*_i_* was calculated using the spatial statistics tool in ArcGIS 9.0 Arc Tool Box.

As the point data available for this study were digitized from block-level maps contemporary to the original outbreaks, the city block was the finest resolution available for spatial statistics. The city block shape in Washington, D.C. was, and still is, complicated and not comprised of only simple geometric squares or rectangles. Several blocks were triangular or irregular in shape. The NPS grid tool only develops symmetrical grid cells. To develop a grid surface that best represented this irregular network of block shapes, a systematic measurement survey was performed within the GIS to calculate the average block size. First, the city was divided into it four primary sections (Northwest, Northeast, Southwest, Southeast) to insure that all parts of the city were included in the grid cell size determination (Figures 2 and 4). Measurements of length and width were collected for 25 square or rectangular blocks in each of the four parts that best represented that section of the city. The mean length and width were calculated for the 100 measured blocks and used as the grid cell size.

The number of cases occurring in each grid cell were summated using the Count Points in Polygon Tool available in the Hawth's Anaylsis Tools extension for ArcGIS 9.0 [45].

To determine at what scale clusters appeared during the 1906 to 1909 typhoid outbreaks, multiple distance values were used in this study. The distances *(d) *were set to 150, 250, 500, 750 and 1000 meters. The smallest distance, 150 m, was selected to capture localized infections, such as residences clustered around a single shared water source. The largest distance, 1000 m, was selected to capture larger outbreaks more representative of a global infection source, such as the city-wide water supply. As the *G*_i_* values are normal variants of the z-distribution, only those *G*_i_* values greater than 2.0 were considered significant, in order to be more conservative than α = 0.05. Following Getis et al. [15] the highest *G*_i_* value for every grid cell was considered the peak of the typhoid cluster. In this way, although a cluster at 500 m may have a *G*_i_* value exceeding 2.0 at the 500 m distance, if the *G*_i_* value at 150 m exceeded that *G*_i_* at 500 m, the 500 m distance was not counted as being significant. In other words, for a grid cell to remain a member of a statistically significant cluster from one distance to another, the *G*_i_* value must increase from test distance size to test distance size. If the *G*_i_* value did not increase with distances, though values may have been greater than 2.0, they were not considered members of clusters. This is defined as the critical distance, *d*_*c *_in Getis and Aldstadt [46]. All clusters presented in this study are defined at *d*_*c*_.

Maps were produced from significant clusters for each year, 1906–1909.

To test whether typhoid outbreak regions were temporally stable across the study period, a summary program was developed in SAS v9.0 to tabulate the total number of years (1 – 4) that a grid cell was a member of a significant cluster at each given distance value. Additionally, we evaluated the total number of years any given grid cell was significant regardless of *d*_*c*_. Temporal stability of the smallest clusters was recorded in order to determine whether single "culprit" locations, such as a shared well, were systematically causing infection.

## Competing interests

The author(s) declare that they have no competing interests.

## Authors' contributions

This study was conceived by SEH. SEH developed the data sets and produced the GIS layers for analysis. SEH and JKB developed the spatial grids methodology, and performed the Ripley's K-function and *G*_i_* statistic. JKB produced the SAS program for data reduction and data summary. All three authors participated in the production and final review of the text.
